# Priming the Pump: Preparing the Advanced Practitioner in Oncology for Scientific Innovation

**Published:** 2017-07-01

**Authors:** Sandra Kurtin

**Affiliations:** Founding Board Member and Treasurer for APSHO Conceptual Originator of Priming the Pump Initiative

The unprecedented, almost frenetic pace of scientific innovation relative to the diagnosis and treatment of cancer demands a level of continuous engagement on the part of oncology providers to safely and effectively integrate these changes into practice. There have been 209 US Food and Drug Administration (FDA) approvals in the past decade for either new oncology drugs or new indications for existing drugs, and 55 of these indications (26%) were approved in the past 2 years. The number of phase II/III trials with new molecules or combinations of molecules suggests this pace of approvals will continue or potentially increase in the coming years. This trend and tempo of continuous discovery and drug approval emphasize the need for innovative educational strategies to support oncology providers.

The recent elucidation of targets and pathways that may be exploited for therapeutic benefit and the subsequent ability to then develop disease-modifying agents that effectively manipulate these targets and pathways in ways that improve disease control, and in some cases overall survival, are profound. Combining newer targeted agents, immune-mediating agents, and existing antineoplastic agents to achieve maximal therapeutic benefit while mitigating treatment-related adverse events is at the core of emerging treatment paradigms. Many of these agents, although investigated initially in one or two disease states, have subsequently been studied in multiple disease states that share a common pathway or target.

The number of agents that have therapeutic indications for both hematologic and solid malignancies is growing. Therefore, oncology providers, including advanced practitioners, must obtain and maintain knowledge about not only the disease state, but the pathways and targets that are active and might be manipulated for therapeutic benefit in each one. In addition, knowledge relative to the normal function of these pathways and targets, to factors that lead to development of malignancies, and to the off-target effects of novel therapies will be necessary to understand and mitigate disease or treatment-related adverse events.

## WHAT IS PRIMING THE PUMP?

Priming the Pump (PTP) is an educational and practice initiative developed by the Advanced Practitioner Society for Hematology and Oncology (APSHO) aimed at creating and maintaining an intuitive and interactive foundation of knowledge relative to emerging therapies. By using the underlying principles of disease states, pathways, targets, and patient factors, a mix of online and print resources are being developed. These resources allow advanced practitioners in oncology to build a current and clinically relevant scope of knowledge to facilitate more effective integration of new therapeutic agents used in the clinical management of patients with cancer.

The content is being developed by interdisciplinary teams with expertise in each disease state or therapeutic area (e.g., pathways/targets). Supplements and resources published in conjunction with the *Journal of the Advanced Practitioner in Oncology* (JADPRO) are appearing this year in print and digital formats. The initial series will focus on non–small cell lung cancer, chronic lymphocytic leukemia, and melanoma, with in-depth discussion of the key pathways, targets, and therapeutic agents for each disease state. Strategies for clinical management and support of patients with each of these diseases using these new agents will be provided.

The first installment of this series, a JADPRO supplement entitled *Understanding the Role of Targets and Pathways in the Treatment of Non–Small Cell Lung Cancer*, will be published this month. Look for a video roundtable discussion to be available on advancedpractitioner.com and APSHO.org soon.

This is an exciting time in oncology practice, and the future is bright. However, achieving the outcomes obtained in clinical trials responsible for the approval of these new drugs requires emulation of the clinical trials process as closely as possible. In collaboration with the members of the PTP Task Force and Education Committee, APSHO and JADPRO are pleased to begin the process for developing tools and strategies to meet this challenge.

